# Emergency department presentations for deliberate self-harm and suicidal ideation in 25–39 year olds following agency-notified child maltreatment: results from the Childhood Adversity and Lifetime Morbidity (CALM) study

**DOI:** 10.1017/S2045796024000192

**Published:** 2024-03-27

**Authors:** S. Kisely, C. Bull, M. Trott, U. Arnautovska, D. Siskind, N. Warren, J. Moses Najman

**Affiliations:** 1 Princess Alexandra Hospital Southside Clinical Unit, Greater Brisbane Clinical School, Medical School, The University of Queensland, Woolloongabba, QLD, Australia; 2 Departments of Psychiatry, Community Health and Epidemiology, Dalhousie University, Halifax, NS, Canada; 3 Metro South Addiction and Mental Health Service, Brisbane, QLD, Australia; 4 School of Public Health, The University of Queensland, Herston, QLD, Australia; 5 School of Social Sciences, The University of Queensland, St Lucia, QLD, Australia

**Keywords:** birth cohort, child maltreatment, deliberate self-harm, linked data, suicidal ideation

## Abstract

**Aims:**

To compare prospective reports of child maltreatment (CM) with emergency department (ED) presentations for deliberate self-harm (DSH) and suicidal ideation in individuals aged between 25 and 39 years old.

**Methods:**

Linked records between the Mater-University of Queensland Study of Pregnancy birth cohort and Queensland administrative health data were used, which included notifications to child protection agencies for CM. ED presentations for individuals aged between 25 and 39 years of age for suicidal ideation, suicidal behaviour or poisoning by paracetamol or psychotropic medications where the intention was unclear were examined using logistic regression analyses.

**Results:**

A total of 609 (10.1%) individuals were the subject of one or more CM notifications for neglect or physical, sexual or emotional abuse before the age of 15 years. Of these, 250 (4.1%) presented at least once to ED for DSH and/or suicidal ideation between 25 and 39 years of age. In adjusted analysis, any notification of CM was associated with significantly increased odds of presenting to ED for these reasons (aOR = 2.80; 95% CI = 2.04–3.84). In sensitivity analyses, any notification of CM increased the odds of the combined outcome of DSH and suicidal ideation by 275% (aOR = 2.75; 95% CI = 1.96–4.06) and increased the odds of DSH alone by 269% (aOR = 2.69; 95% CI = 1.65–4.41).

**Conclusions:**

All CM types (including emotional abuse and neglect) were associated with ED presentations for DSH and suicidal ideation in individuals between 25 and 39 years of age. These findings have important implications for the prevention of DSH, suicidal ideation and other health outcomes. They also underscore the importance of trauma-informed care in ED for all individuals presenting with DSH and suicidal ideation.

## Introduction

Suicidal behaviour varies in severity from suicidal ideation (i.e., thoughts about taking one’s own life) through to actions with a serious intent to die (Harmer *et al.*, 2023; Molnar *et al.*, 2001). One pervasive risk factor for both suicidal ideation and behaviour is child maltreatment (CM) (Angelakis *et al.*, 2019; Van Orden *et al.*, 2010). CM comprises ‘… *physical, sexual and emotional abuse, and neglect inflicted upon a child by a person responsible for their care and wellbeing*’ (Australian Institute of Health and Welfare, 2022). The relationship between CM and suicidal behaviour is multifaceted and complex. Interactions between genetics, biology (e.g., the hypothalamic–pituitary–adrenal axis), psychology and sociocultural environments all play a part (Andreassen *et al.*, 2023; Berardelli *et al.*, 2020; Costanza *et al.*, 2022; Mann *et al.*, 2004; Paquola *et al.*, 2016).

To date, the focus of epidemiological research has largely been on sexual abuse and physical abuse where there are strong associations with subsequent suicide attempts (Angelakis *et al.*, 2019; Ng *et al.*, 2018; Zatti *et al.*, 2017). Comparably, fewer studies have examined associations between suicidality and emotional abuse or neglect, though there is still some evidence of strong relationships (Angelakis *et al.*, 2019; Zatti *et al.*, 2017). One limitation of the literature is that most information comes from cross-sectional or longitudinal studies of adults who retrospectively self-report CM (Angelakis *et al.*, 2019; Lawrence *et al.*, 2023; Zatti *et al.*, 2017). An example is the Australian Child Maltreatment Study (ACMS) – a large, cross-sectional, retrospective study of self-reported CM in a sample of 8,503 Australians aged 16 years and older (Lawrence *et al.*, 2023). The findings from these studies may, therefore, be limited by recall bias, the use of clinical as opposed to population samples and the possibility that CM could be both a cause and an effect of deliberate self-harm (DSH) (Fergusson *et al.*, 2000; Steele *et al.*, 2024; Widom *et al.*, 2004). In the most comprehensive systematic review of the field, only 8 (of 68) studies reporting prospective CM notifications from objective records were identified (Angelakis *et al.*, 2019). Five of those used independent records rather than questionnaires or interviews for information on CM, and one investigated agency-reported CM but failed to consider CM subtypes (Brown *et al.*, 1999). A subsequent study using forensic medical records to identify CM was restricted to sexual abuse (Papalia *et al.*, 2023). Thus, despite being a more objective measure of CM incidence, prospective studies are less common.

Our previous work from the Mater-University of Queensland Study of Pregnancy (MUSP) followed up a birth cohort to age 30 in an attempt to address some of these issues (Kisely *et al.*, 2022b). Of the original 7,223 members of the birth cohort (Najman *et al.*, 2015), 7,214 had data that could be linked to notifications of CM from Queensland-wide statutory agencies (up to 15 years of age). At the 30-year follow-up point, 12.1% (*n* = 304) disclosed that they had self-harmed at some time in their lives, and 150 (4.2%) said they had wanted to die (Kisely *et al.*, 2022b). In adjusted analysis, agency-reported CM demonstrated significant associations with both self-harm and wanting to die. Indeed, these associations were greatest where physical and emotional abuse had been reported. However, loss to follow-up was a significant limitation of this study, with only one-third (*n* = 2,427) of the birth cohort remaining in the study by 30 years of age. Attrition was highest in participants from socioeconomically disadvantaged backgrounds, potentially introducing bias in the results. To overcome these potential biases, we linked administrative health data to the MUSP birth cohort data. Thus, the aim of this study was to compare prospective reports of CM (up to 15 years of age) in the MUSP birth cohort with emergency department (ED) presentations for DSH and/or suicidal ideation between 25 and 39 years old using linked administrative data from the whole cohort, not just those who were followed up. We hypothesised strong associations between ED presentations for DSH and/or suicidal ideation and all forms of CM.

## Methods

We report results from the Childhood Adversity and Lifetime Morbidity study (Kisely *et al.*, 2023). This was a data linkage study using data from the MUSP birth cohort (Najman *et al.*, 2015) and Queensland-wide administrative health datasets (Kisely *et al.*, 2023). The linkage enabled us to compare ED presentations for DSH and/or suicidal ideation in participants who had experienced agency-reported CM compared to those who had not (Kisely *et al.*, 2023).

We received ethics approval from the University of Queensland Human Research Ethics Committee (HREC) (2021/HE001925) and the Metro South Health HREC (HREC/2022/QMS/83690). We prospectively registered a pre-trial protocol with the Australian and New Zealand Clinical Trials Registry (ACTRN12622000870752) (Kisely *et al.*, 2023). Reporting of this study followed STrengthening the Reporting of OBservational studies in Epidemiology guidance (Von Elm *et al.*, 2008) and the REporting of studies Conducted using Observational Routinely collected health Data statement (Benchimol *et al.*, 2015).

### Participants

Detailed methods of MUSP methods are described elsewhere. In brief, the MUSP study recruited participants who were born at the Mater Mothers Hospital, the principal obstetrics unit for Brisbane, between 1981 and 1983 (Najman *et al.*, 2015). In September 2000, notifications of CM to the Department of Families, Youth and Community Care were anonymously linked to the longitudinal database (such that no personal details were released to the researcher undertaking the analyses) for 7,214 individuals in the birth cohort (Strathearn *et al.*, 2020). Reports were substantiated if, after investigation, there was a reasonable belief of CM across any of the four subtypes: physical, sexual or emotional abuse and/or neglect. Notifications were recorded between the ages of 0 and 15 years old.

### Health data sources

The MUSP cohort was anonymously linked to the Queensland Emergency Data Collection (EDC) from 2008 to 2020 using encrypted identifiers (Kisely *et al.*, 2023). Resultantly, the minimum age of individuals presenting to the ED was 25 years old, and the maximum age was 39 years old. We used the most recent episode to identify cases in each database. The EDC uses a limited range of International Classification of Diseases, 10th Revision, Austrlian Modification (ICD-10-AM) diagnoses (Sveticic *et al.*, 2020). DSH is only coded as X84 (‘intentional self-harm by unspecified means’) even though these presentations are covered by a range of codes between X60 and X84. Thus, we used a broad definition of ICD-10-AM codes describing DSH and suicidal ideation, including DSH (X84), suicidal ideation (R45.81) and poisoning by paracetamol (T39.1), benzodiazepines (T42.4), antidepressants (T43.9) or antipsychotic medications (T43.5). This broad definition arose from previous research using the EDC where the authors demonstrated that reliance on ICD-10-AM codes X84 and R45.81 alone missed a large proportion of relevant cases (Sveticic *et al.*, 2020). However, we also conducted sensitivity analyses of the effect of restricting to the outcomes to either X84 or R45.81 and, lastly, to X84 alone.

### Data linkage and quality

**Figure 1.**
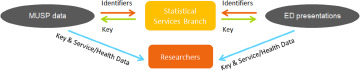
Depiction of database linkage performed by the Statistical Services Branch (SSB) of Queensland Health.

Data linkage was performed by the Statistical Services Branch (SSB) of Queensland Health (Kisely *et al.*, 2023). The MUSP data custodian provided a dataset containing only the names, birth dates and sex of the birth cohort participants to the SSB (Fig. 1). Within the SSB, Oracle software was used to match these details with the Queensland Health Master Linkage File (MLF). The SSB subsequently assigned a unique key to each linked record and shared this with the EDC data custodian, who used it as an identifier for the records that were sent to the researchers stripped of any personal details. Additional information on the accuracy and quality of data linkage is available via the Queensland Data Linkage Framework (State of Queensland (Queensland Health), 2020).

### Statistical analysis

Data were first described using counts and frequencies. Using logistic regression, we measured bivariate associations between all forms of notified and substantiated CM (aggregate and per subtype) and ED presentations for broad definition DSH and/or suicidal ideation. As ED presentations were strongly skewed to the right, we dichotomised these into ‘never’ versus ‘ever occurred’. Logistic regression models were then adjusted for several variables given their association with either CM or psychiatric symptoms in our previous research (Kisely *et al.*, 2020). These included gender, parental race, parental relationship status and family income at the time of study entry. This ensured covariates predated any subsequent maltreatment.

We also performed sensitivity analysis by using propensity scores in adjusted logistic regression models. Here, we included gender, parental race, parental relationship status and family income at the time of study entry as covariates and additionally included propensity scores per variable that represented the entire MUSP cohort (*n* = 7,214), not just those whose administrative health data could be linked (*n* = 6,087). This approach was used as opposed to undertaking multiple imputations because we could not assume that the data were missing at random. Finally, as noted above, we performed sensitivity analyses restricted to the combined outcome of X84 and R45.81, and X84 alone.

## Results

**Figure 2.**
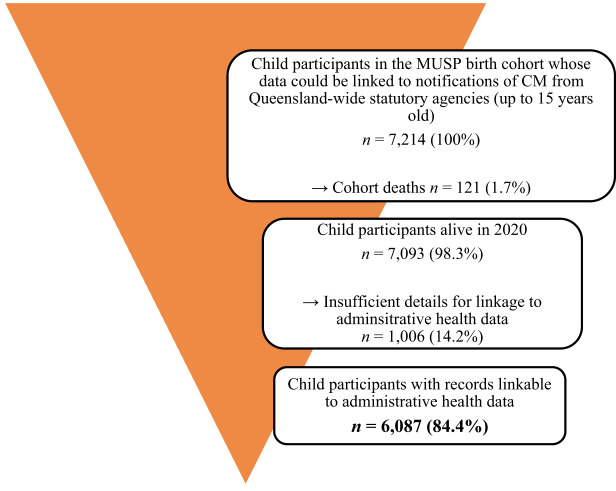
Study data flow diagram.

Of the 7,214 participants, 121 died over the study period and 1,006 had insufficient details (e.g., no first name) to search for potential matches in the Queensland Health MLF (Fig. 2). Those with missing details were significantly more likely to be from an Indigenous background (OR = 2.26; 95% CI = 1.80–2.83), have had parents who were not living together (OR = 1.57; 95% CI = 1.32–1.87) or were on a low income at baseline (OR = 1.67; 95% CI = 1.46–1.92). Resultantly, 6,087 participants had records that could be linked; 51.6% were male (*n* = 3,143) and 5% (*n* = 326) Indigenous Australians (see Table 1 for full demographic details).

**Table 1.** Variables associated with ED presentations for individuals aged between 25 and 39 years old for broad definition DSH and/or suicidal ideation^a^

	ED presentations for broad definition DSH and/or suicidal ideation						
	Never (*n* = 5,837)		Ever occurred (*n* = 250)		Significance		
	Count	%	Count	%	OR (95% CI)	Adj OR (95% CI)^b^	*P*
Gender (male)	3,002	51.4%	141	56.4%	1.22 (0.95−1.57)	1.26 (0.97−1.63)	0.08
Identified as Indigenous	309	5.3%	14	5.6%	1.06 (0.61−1.84)	0.88 (0.50−1.54)	0.67
Parents not living together at birth	733	12.6%	45	18.0%	1.53 (1.10−2.13)	1.29 (0.91−1.82)	0.15
Family income prior to birth ≤$10,399/year	1,754	30.0%	86	34.4%	1.22 (0.94−1.59)	1.28 (0.91−1.82)	0.45
Presence of any CM notifications	551	9.4%	58	23.2%	2.90 (2.13−3.94)	2.80 (2.04−3.84)	**<0.001**
Presence of any CM substantiated notifications	353	6.0%	36	14.4%	2.61 (1.81−3.78)	2.46 (1.69−3.59)	**<0.001**

ED = emergency department; DSM = deliberate self-harm; OR = odds ratio; CM = childhood maltreatment.

a Broad definition DSH and/or suicidal ideation includes the following ICD-10-AM codes: X84, R45.81, T39.1, T42.4, T43.9, T43.5.

b Variables used in adjustment: baseline gender; parental race; parental relationship; family income at baseline.

Bolded values indicate *p* ≤ 0.05.

### Child maltreatment

Notifications of possible CM were made for 609 (10.0%) of the 6,087 individuals. For 309 participants, notifications were for two or more types of CM. Neglect was the most common type of CM (364 cases; 6.0%), followed by physical abuse (359 cases; 5.9%), emotional abuse (333 cases; 5.5%) and sexual abuse (197 cases; 3.2%). Of these notifications, 389 (6.4%) individuals were confirmed as substantiated cases. Instances of physical abuse were most prevalent (204 cases; 3.4%), followed by emotional abuse (199 cases; 3.3%), neglect (192 cases; 3.2%) and sexual abuse (109 cases; 1.8%).

Notifications of possible CM were more common in participants who were female (52.2% vs 47.9%; OR = 1.20; 95% CI = 1.02–1.42) or Indigenous (10.2% vs 4.8%; OR = 2.27; 95% CI = 1.69–3.03), as well as those whose parents who were not living together at baseline (26.4% vs 11.3%; OR = 2.83; 95% CI = 2.32–3.45) or were on a low income (46.0% vs 28.5%; OR = 2.14; 95% CI = 1.80–2.53).

### Presentations

A total of 250 (4.1%) individuals presented to the ED at least once for the broad definition of DSH or suicidal ideation (Table 1). In unadjusted analysis, participants who had been the subject of any form of notified or substantiated CM were significantly more likely to present with DSH and/or suicidal ideation, as did those whose parents were not living together at the time of their birth. Associations with other sociodemographic variables were non-significant.

Table 2 illustrated significant associations between ED presentations for DSH and/or suicide ideation and all subtypes of notified and substantiated CM. The adjusted odds of presenting to ED were 2.64 (95% CI = 1.62–4.31) where there was a history of substantiated neglect, 3.07 (95% CI = 1.95–4.84) where there was a history of substantiated physical abuse and 3.39 (95% CI = 2.17–5.30) where there was a history of substantiated emotional abuse. While sexual abuse demonstrated the weakest association to ED presentations (aOR = 2.07; 95% CI = 1.03–4.19), it was still statistically significant. These results were replicated in the propensity score analysis, suggesting similarities in participants’ baseline characteristics where administrative health records were and were not able to be linked.

**Table 2.** Associations between CM notifications and ED presentations for individuals aged between 25 and 39 years old for broad definition DSH and/or suicidal ideation^a^

	ED presentations for broad definition DSH and/or suicidal ideation				
	OR (95% CI)	Adj OR (95% CI)^b^	*P*	Propensity Adj OR (95% CI)^b^	*P*
Any agency-reported notifications of CM	2.90 (2.13−3.94)	2.80 (2.04−3.84)	**<0.001**	2.80 (2.04−3.84)	**<0.001**
Physical abuse	3.40 (2.40−4.84)	3.22 (2.24−4.62)	**<0.001**	3.10 (2.15−4.47)	**<0.001**
Emotional abuse	3.60 (2.52−5.15)	3.41 (2.36−4.92)	**<0.001**	3.40 (2.36−4.91)	**<0.001**
Neglect	2.72 (1.87−3.60)	2.55 (1.74−3.74)	**<0.001**	2.57 (1.75−3.76)	**<0.001**
Sexual abuse	2.14 (1.26−3.62)	2.09 (1.22−3.58)	**<0.01**	2.10 (1.23−3.59)	**<0.01**
Two or more types	3.34 (2.37−4.70)	3.17 (2.22−4.50)	**<0.001**	3.20 (2.25−4.55)	**<0.001**
Any substantiated notifications of CM	2.61 (1.81−3.78)	2.46 (1.69−3.59)	**<0.001**	2.47 (1.69−3.61)	**<0.001**
Physical abuse	3.34 (2.14−5.21)	3.07 (1.95−4.84)	**<0.001**	3.08 (1.65−4.85)	**<0.001**
Emotional abuse	3.62 (2.33−5.61)	3.39 (2.17−5.30)	**<0.001**	3.40 (2.18−5.30)	**<0.001**
Neglect	2.86 (1.77−4.63)	2.64 (1.62−4.31)	**<0.001**	2.66 (1.63−4.34)	**<0.001**
Sexual abuse	2.14 (1.07−4.29)	2.07 (1.03−4.19)	**0.04**	2.07 (1.03−4.19)	**0.04**

ED = emergency department; OR = odds ratio; CM = childhood maltreatment.

a Broad definition DSH and/or suicidal ideation includes the following ICD-10-AM codes: X84, R45.81, T39.1, T42.4, T43.9, T43.5.

b Variables used in propensity adjustment: baseline gender; parental race; parental relationship; family income at baseline.

Bolded values indicate *p* ≤ 0.05.

There were 158 presentations for DSH and/or suicidal ideation using just ICD-10-AM codes X84 and R45.81 and 96 for X84 alone. Both showed significant associations with CM of all types except substantiated sexual abuse (Table 3).

**Table 3.** Sensitivity analyses examining associations between CM notifications and ED presentations for individuals aged between 25 and 39 years old for suicidal ideation (ICD-10-AM code R45.81) and/or DSH (ICD-10-AM code X84)

	ED presentations for suicidal ideation and/or DSH					
	Suicidal ideation and DSH			DSH only		
	OR (95% CI)	Adj OR (95% CI)^a^	*P*	OR (95% CI)	Adj OR (95% CI)^a^	*P*
Any agency-reported notifications	2.86 (1.96−4.18)	2.75 (1.86−4.06)	**<0.001**	2.91 (1.80−4.67)	2.69 (1.65−4.41)	**<0.001**
Physical abuse	3.64 (2.38−5.56)	3.41 (2.21−5.27)	**<0.001**	3.55 (2.08−6.06)	3.20 (1.84−5.55)	**<0.001**
Emotional abuse	3.60 (2.33−5.57)	3.38 (2.16−5.28)	**<0.001**	3.58 (2.07−6.19)	3.25 (1.85−5.71)	**<0.001**
Neglect	2.35 (1.45−3.81)	2.16 (1.32−3.54)	**<0.01**	2.29 (1.24−4.23)	2.02 (1.08−3.79)	**0.03**
Sexual abuse	2.55 (1.39−4.67)	2.57 (1.38−4.78)	**<0.01**	2.40 (1.09−5.25)	2.30 (1.04−5.13)	**0.04**
Two or more types	3.26 (2.14−4.97)	3.06 (1.98−4.72)	**<0.001**	3.19 (1.87−5.45)	2.89 (1.66−5.01)	**<0.001**
Any substantiated notifications	2.87 (1.85−4.46)	2.70 (1.72−4.24)	**<0.001**	3.01 (1.74−5.20)	2.73 (1.57−4.80)	**<0.001**
Physical abuse	3.70 (2.19−6.25)	3.37 (1.97−5.76)	**<0.001**	3.89 (2.04−7.40)	3.44 (1.78−6.67)	**<0.001**
Emotional abuse	3.81 (2.25−6.43)	3.55 (2.08−8.05)	**<0.001**	3.99 (2.09−7.61)	3.60 (1.87−6.95)	**<0.001**
Neglect	2.88 (1.60−5.18)	2.63 (1.45−4.77)	**<0.01**	3.71 (1.89−7.26)	3.28 (1.65−6.51)	**<0.001**
Sexual abuse	2.23 (0.96−5.16)	2.22 (0.95−5.19)	0.07	1.79 (0.56−5.74)	1.69 (0.52−5.50)	0.38

ED = emergency department; DSH = deliberate self-harm; OR = odds ratio; CM = childhood maltreatment.

a Variables used in adjustment: baseline gender; parental race; parental relationship; family income at baseline.

Bolded values indicate *p* ≤ 0.05.

## Discussion

Most of the information on the association between CM and DSH and/or suicide ideation comes from cross-sectional or longitudinal studies where reports of abuse are retrospective (Angelakis *et al.*, 2019; Castellví *et al.*, 2017; Ng *et al.*, 2018; Zatti *et al.*, 2017). Moreover, the focus of most previous research has been on physical and sexual abuse rather than emotional abuse or neglect (Angelakis *et al.*, 2019; Castellví *et al.*, 2017; Zatti *et al.*, 2017). Many of these studies have relied on self-reported DSH and/or suicide ideation rather than objective behaviour such as presentation to health services. To our knowledge, this is one of a limited number that (i) examines the long-term effects of prospective agency-reported and substantiated CM on DSH and suicidal behaviour; (ii) includes an adult population and (iii) concerns itself with all forms of CM (physical abuse, emotional abuse, sexual abuse and neglect).

Methodological differences may explain some of the discrepancies in findings between the current study and our previous findings on self-reported self-harm and suicidal ideation (Kisely *et al.*, 2022b). Notably, the prevalence of combined DSH and/or suicide ideation in the current study was 4.1% compared to 12.1% for self-harm and 4.2% for suicidal ideation in our earlier study. Our previous research only showed a consistent association between physical and emotional abuse and self-reported suicidal behaviour, while findings for neglect and sexual abuse were mixed, possibly because the study was under-powered. Results from the ACMS similarly reinforce this discrepancy (Lawrence *et al.*, 2023). ACMS researchers identified that individuals who self-reported CM had 3.93 higher odds of self-harming in the previous 12 months compared to those who had not experienced CM (after adjustment) (Lawrence *et al.*, 2023). They also reported 4.56 times higher odds of attempting suicide (Lawrence *et al.*, 2023). These results are notably higher than findings of the current study.

An advantage of the current study is, therefore, that with greater numbers from both the birth cohort and administrative health data, we could show that all forms of CM had significant associations with common mental disorders that were of sufficient severity to result in an ED presentation. We also identified that emotional abuse and neglect are equally important contributors to subsequent suicidal ideation and behaviour in addition to physical and sexual abuse. Our results are consistent with findings from retrospectively collected data using the Childhood Trauma Questionnaire where emotional abuse and neglect played a significant role in the aetiology of non-suicidal self-injury above and beyond symptoms of depression and anxiety (Brown *et al.*, 2018). Combined, these findings suggest that emotional abuse and neglect may be as important as physical and sexual abuse to DSH and suicidal ideation.

There may be several explanations for our findings. Biologically, CM can effect brain development and neurobiological systems, potentially altering an adult’s ability to regulate mood and stress, thereby increasing vulnerability to suicidal behaviour (Paquola *et al.*, 2016). Genetics may also play a part. Research from the last decade shows associations between genetic variants and major psychiatric disorders (including suicidality), though the mechanistic understanding of these associations is still incomplete (Andreassen *et al.*, 2023). Psychologically, CM can have a lasting impact on an individual’s self-esteem and self-worth (Costanza *et al.*, 2022; Mann *et al.*, 2004). As a result, they are at greater risk of psychiatric or substance use disorders, both of which can lead to suicidal behaviours (Kisely *et al.*, 2021, 2022b). Socially, CM is associated with lower educational attainment and dysfunctional relationships leading to unstable employment or loneliness and a greater risk of suicidal behaviour.

A key strength of this study was the use of state-wide ED records. This reduced the effects of attrition and reporting bias that has limited the generalisability of previous studies using the MUSP cohort (Kisely *et al.*, 2022a; Strathearn *et al.*, 2020). Only ED presentations from 2008 to 2020 were included, thereby eliminating the possibility that presentations preceded any CM notification. Additionally, the minimum age of individuals in the dataset in 2008 was 25 years old, thus lessening the possibility that poisoning with agents such a paracetamol was accidental (Caravati, 2000; Penna and Buchanan, 1991). Use of ED presentations as the primary outcome is a further strength as our previous assessments of suicidal behaviour in MUSP participants were restricted to two self-reported items (Kisely *et al.*, 2022b).

As outlined in the protocol, there are several limitations to this study (Kisely *et al.*, 2023). One is that reliance on agency-notified CM is likely to significantly underestimate the true prevalence of maltreatment particularly as it reflects reporting practices in the 1980s and 90s (Gilbert *et al.*, 2009). Furthermore, even fewer reports were substantiated following investigation by child protection services, further compounding the underestimation. Secondly, the outcomes were derived from administrative data that may be affected by recording bias. For example, other work has suggested that reliance on ICD-10-AM codes R45.81 and X84 alone underestimates the true prevalence of these presentations (Sveticic *et al.*, 2020). Although we used a broad definition encompassing poisoning as the primary outcome, as partly suggested in earlier research, we may not have captured the true number of cases (Sveticic *et al.*, 2020). We were also unable to include as covariates sociodemographic data at the time of ED presentation because of missing data. Furthermore, the data do not encompass individuals with undiagnosed or untreated disorders or those who received outpatient treatment. We were also unable to determine whether the 121 participants who died over the study period did so because of suicide. Additionally, about 14% of the MUSP cohort lacked sufficient information for linkage to administrative health data. On the other hand, associations were unaltered using propensity score analyses of adding a variable representing possible baseline confounders across the whole MUSP cohort. Finally, we cannot exclude the possibility that the lack of an ED record was due to a failure to find a linkage to the administrative database rather than that the absence of an ED presentation. However, given the strength of association in our findings, this is unlikely to have greatly affected the overall results.

The findings of the current study have important implications for child protection, the primary and secondary prevention of DSH and suicidal ideation, as well as care practices in ED. Early identification, escalation and intervention for the primary prevention of CM remain critically important. Intervening at the earliest possible juncture where CM is taking place may lead to drastic reductions in mental illness in the future. Despite its complexities, this remains an area where greater research is warranted. Given the clear link between each type of CM and DSH or suicidal ideation, it is important that preventive programmes consider CM from more than just the perspective of physical and sexual abuse. Moreover, greater research is required to further verify the impact of emotional abuse and neglect not only on DSH and suicidal ideation across the life span but other mental health outcomes, suicide attempts and deaths by suicide. These findings also highlight the importance of trauma-informed care for people presenting to the ED for DSH and/or suicidal ideation.

## Data Availability

Due to privacy, ethical and legal considerations, the administrative health data cannot be shared without direct approval from relevant data custodians and the Office of Research and Innovation of Queensland Health. Contact details for Queensland Health custodians can be found at https://www.health.qld.gov.au/__data/assets/pdf_file/0034/843199/data_custodian_list.pdf.
