# Emergency department presentations for deliberate self-harm and suicidal ideation in 25–39 year olds following agency-notified child maltreatment: results from the Childhood Adversity and Lifetime Morbidity (CALM) study – CORRIGENDUM

**DOI:** 10.1017/S204579602400026X

**Published:** 2024-04-12

**Authors:** S. Kisely, C. Bull, M. Trott, U. Arnautovska, D. Siskind, N. Warren, J. Moses Najman

DOI: https://doi.org/10.1017/S2045796024000192, Published by Cambridge University Press 27 March 2024

The original article contained an error in the title. The error has been corrected and the article republished.

The authors apologise for the error.
